# Electrolytic ammonia removal and current efficiency by a vermiculite-packed electrochemical reactor

**DOI:** 10.1038/srep41030

**Published:** 2017-01-19

**Authors:** Liang Li, Ji Yao, Xueyou Fang, Yuanxing Huang, Yan Mu

**Affiliations:** 1 School of Environment and Architecture, University of Shanghai for Science and Technology, Shanghai 200093, China; 2 Hebei Province Environmental Monitoring Center, Shijiazhuang 050056, China

## Abstract

The ammonia removal as well as the current efficiency during electrolysis was investigated by using a vermiculite-packed electrochemical reactor under continuous mode. Experimental results showed that adsorption of ammonia by vermiculite and electrolytic desorption of ammonia simultaneously existed in the reactor, leading to 89% removal of initial 30 mg N/L ammonia and current efficiency of 25% under the condition of 2.0 A, 6.0 min hydraulic retention time with 300 mg Cl/L chloride as the catalyst. The ammonia removal capacity had a linear relationship with the products of hydraulic retention time, current and chloride concentration within experimental conditions. The treatment results of secondary effluent indicated that 29.9 mg N/L ammonia can be reduced to 4.6 mg N/L with 72% removal of total nitrogen and a current efficiency of 23%, which was 2% less than synthetic wastewater due to the reducing components in the real wastewater.

Water is an essential and even irreplaceable resource for the social and economic sustainable development. Around the world, water scarcity happens in the past few decades in the forms of both quantity shortage and low quality originated from various types of pollution. Functional disability of surface water bodies is brought from certain contaminants similar to the relationship between eutrophication and extra nutrient input.

Ammonia, one of the main nitrogenous pollutants in wastewater, has an adverse impact on natural water environment due to its characteristics of bad smell, aquatic biota toxicity^1^, conducive to eutrophication^2^, and oxygen-consuming nature in nitrification process. The main sources of ammonia include the decomposition of nitrogenous organic compounds in urine or feces, coking or synthetic ammonia industrial effluents, extra fertilizer from agriculture etc. Both ammonia molecular (NH_3_) and ammonium ion (NH_4_^+^) exist in aqueous phase, and transform to each other depending on pH^3^. The displacement of K^+^ by elevated NH_4_^+^ was found to be the reason for ammonia’s toxicity to all vertebrates causing convulsions, coma and death^4^. The ammonia inhibition of microbial activity strongly correlated with both the total amount of ammonia and the system pH in biological wastewater treatment process^5^,^6^. Moreover, ammonia content has a positive relationship with the bacterial growth in drinking water grid^7^,^8^.

For those reasons above, the removal of ammonia is required and high standard is set (<5 mg N/L in China) for wastewater discharge. Various means have been investigated for different purposes, including chloride-mediated electrolysis^9^, breakpoint chlorination^10^, ion exchange^11^, supercritical oxidation^12^, membrane filtration^13^, biological treatment^14^, air stripping^15^, chemical precipitation^16^, adsorption^17^,^18^,^19^ etc.

Among those techniques, electrolysis has attracted more and more attention due to its effectiveness, temperature independence, no secondary pollution, easy automatic control and maintenance etc^20^. New electrode materials could lead to improved electrochemical activities. For example, improved generation of H_2_O_2_ was observed by doped mesoporous carbon and graphene cathodes, respectively^21^,^22^. Moreover, pollutant removal and current efficiency strongly depends on the mass transfer process in the aqueous phase. Recently, various materials have been investigated in electrolytic system as the packing medium, which include granular activated carbon^23^, graphite^24^, zeolite^25^, modified bentonite^26^, foaming nickel^27^ etc. Enhanced removal of contaminants as well as decreased energy requirement were observed, which can be ascribed to large surface area and active sites, improved conductivity, high mass transfer driven by adsorption and so on.

Vermiculite, a secondary metamorphic mineral consisted of magnesium, iron and aluminum silicate, is usually formed by weathering or hydrothermal alternation of biotite or phlogopite^28^. Promising ammonia adsorption was found with vermiculite and its composite with BaCl_2_ named “porous matrix with active salt”^29^. Moreover, high adsorption capacity and synergistic effect with biodegradation was once observed during removal of Di-(2-ethylhexyl) phthalate^30^.

As far as we know, vermiculite has not been used as the packing material for electrolysis cells. This research aimed to investigate the possibility of electrolytic removal of ammonia in electrolysis cells packed with vermiculite. Possible products and affecting factors were tested, which further led to the validation of pathways and mechanisms for ammonia removal. Current efficiencies were also calculated based on the main products.

## Results and Discussion

### Characterization of vermiculite

**Figure 1:**
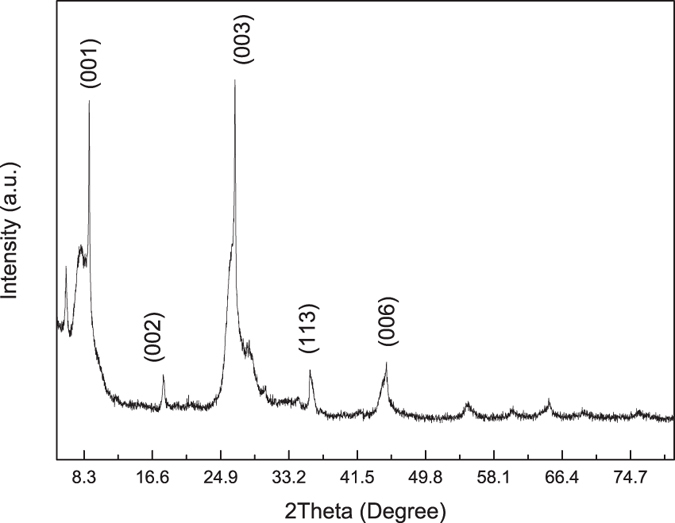
XRD of the vermiculite packed in the electrolysis cell.

Before packed into the electrolysis cell, the as received vermiculite sample was characterized by XRD as shown in Fig. 1. The XRD pattern shows that five intensive diffraction peaks at 2θ of 8.9°, 17.9°, 26.6°, 35.8° and 45.1° were indexed to (001), (002), (003), (113) and (006) diffraction planes, which is characteristic of phlogopite (JCPDS 10-0495) relating to the characteristics of interlayer distance of 9.9, 5.0, 3.3, 2.5 and 2.0 Å, respectively. This result was similar to Deng *et al*., in which two strong diffraction peaks at 2θ = 8.7° and 27.5° were observed for expanded vermiculite^31^. Another low-intense diffraction peak is found nearly at 6.1° and assigned as (001) diffraction plane of vermiculite (JCPDS 74-1732) with the basal spacing of 14.4 Å, similar with natural vermiculite characterized by Yu *et al*.^32^. The changes in the XRD reflection positions reflect the size of the hydrated metal cations and organic cations in the interlayer of vermiculite^33^. The packed vermiculite used in this research is mainly composed of vermiculite and vermiculite-phlogopite mixed layer minerals.

**Figure 2:**
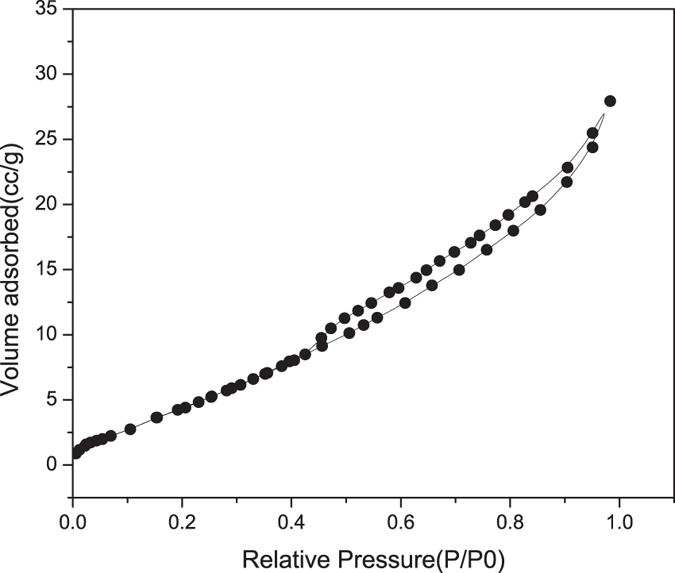
Nitrogen adsorption-desorption curve of the vermiculite.

Nitrogen adsorption-desorption isotherms of vermiculite was depicted in Fig. 2, which can be ascribed to type IV according to the IUPAC classification with small hysteresis loop of a capillary condensation in the mesopores^34^. This point can be verified by the average pore size of 6.5 nm calculated using the DFT method from desorption data. The BET surface area was 37.2 m^2^/g, which was higher than Yunli vermiculite (Xinlong Vermiculite Co. Ltd., Yunli, Xinjiang, China) of 5 m^2^/g, but much smaller than the acid modified samples of 498–764 m^2^/g^35^. The average pore volume was 0.043 cm^3^/g, which was larger than that of 0.029 cm^3^/g reported by Yu *et al*.^32^.

### Mechanism and pathway for ammonia removal

**Figure 3:**
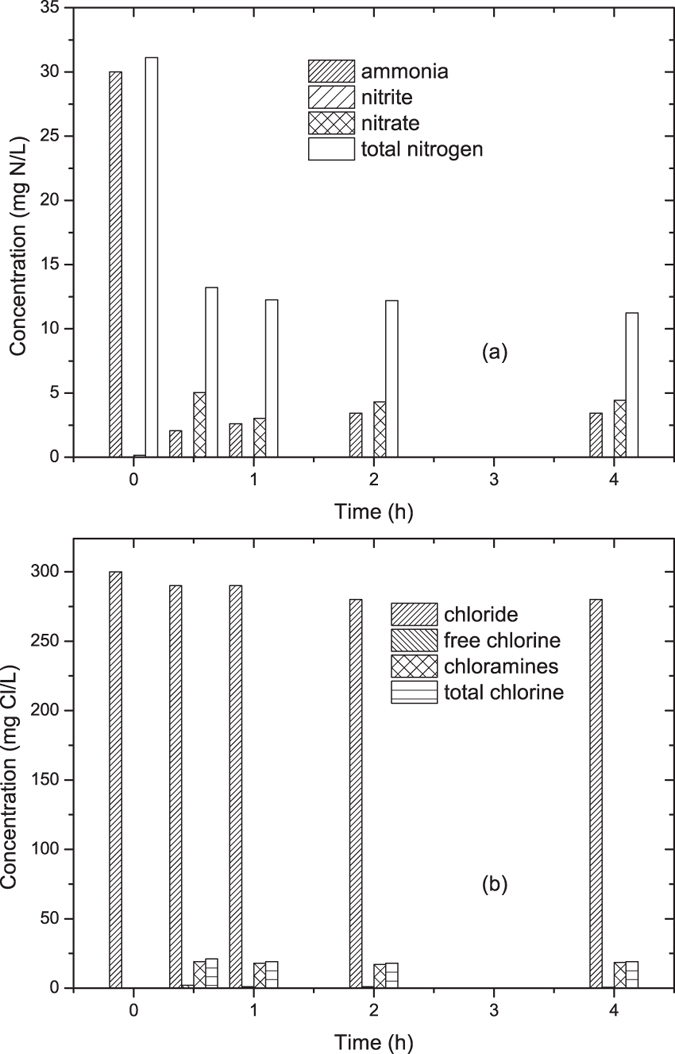
The transformation of different elements during electrolysis at 2.0 A and 6.0 min HRT: (**a**) nitrogen; (**b**) chlorine.

Similar to the actual wastewater, the influent ammonia concentration was adjusted to 30.0 mg N/L by adding (NH_4_)_2_SO_4_ into de-ionized water. Through electrolysis at a current of 2.0 A and 6.0 min HRT, the ammonia and total nitrogen decreased to 3.4 and 11.2 mg N/L, respectively, while the nitrate concentration increased to 4.4 mg N/L as shown in Fig. 3(a). Among all the ammonia removed, 75% was converted to gaseous nitrogen, and the other 25% still existed in aqueous phase in the form of nitrate (17%) and chloramines (8%), respectively. Nitrite was minimum (<0.01 mg N/L) during the electrolytic process. Moreover, Fig. 3(b) showed a reduction of chloride ion from 300 mg Cl/L to 280 mg Cl/L, together with a generation of 0.5 and 18.5 mg Cl/L free chlorine and chloramines, respectively.

Direct oxidation on anode, indirect oxidation by ·OH and active chlorine were proved to be the main mechanisms for electrolytic removal of ammonia from aqueous phase^20^. With the presence of chloride ion, active chlorine was generated through eqs (1) and (2), and ammonia was mainly removed through indirect oxidation by active chlorine as shown in eqs (3) and (4) 36. Side reactions might happen, and nitrate was generated through eqn. (5). Other products such as monochloramine, dichloramines, trichloramines were generated through eqs (6),(7),(8). Competitive reactions also consumed part of the currents for the decomposition of H_2_O into H_2_ and O_2_. High O_2_-evolution overpotential might be beneficial for ammonia removal.

At the anode:


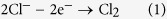


In solution:


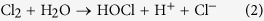



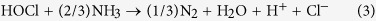



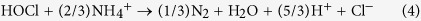



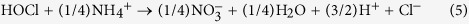



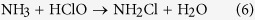



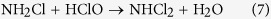



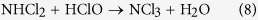


When vermiculite was packed into the reactor, simultaneously adsorption/ion-exchange of ammonia and desorption by active chlorine played an important role in ammonia removal. On one hand, the retention time of ammonia in the electrolysis cell was extended through the adsorption/ion-exchange by vermiculite, which was beneficial for the electrolytic process. On the other hand, vermiculite was kept unsaturated through the simultaneously regeneration by active chlorine. Moreover, accumulation of ammonia and active chlorine on the surface of vermiculite might also help to accelerate the reaction. Similar effect was observed for zeolite packed electrolytic cells^25^. Other packing materials such as activated carbon or foaming nickel had less adsorption of ammonia, and might only contribute for the generation of secondary oxidants.

Current efficiency (CE) of this process can be evaluated based on the main final products of nitrate, nitrogen gas and chloramines as shown in eqn. (9). During electrolysis, 8 mol electrons (z_1_ = 8) were transferred per mol nitrate, while 6 mol electrons (z_2_ = 6) were transferred per mol nitrogen gas. For chloramines, no electron transfer happened considering valence of nitrogen is −3 for monochloramines, dichloramines and trichloramines, which was the same with ammonia. The current efficiency could be calculated to be 25% for the treatment of 30 mg N/L synthetic wastewater under conditions of 2.0 A current and 6.0 min HRT. As more ammonia was retained by vermiculite in the cell, active chlorine was rapidly consumed and converted to chloride ion again. Less current will be used for the decomposition of water, thus the CE was improved.


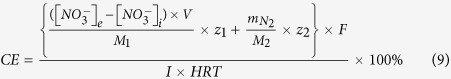


### Influencing factor

**Figure 4:**
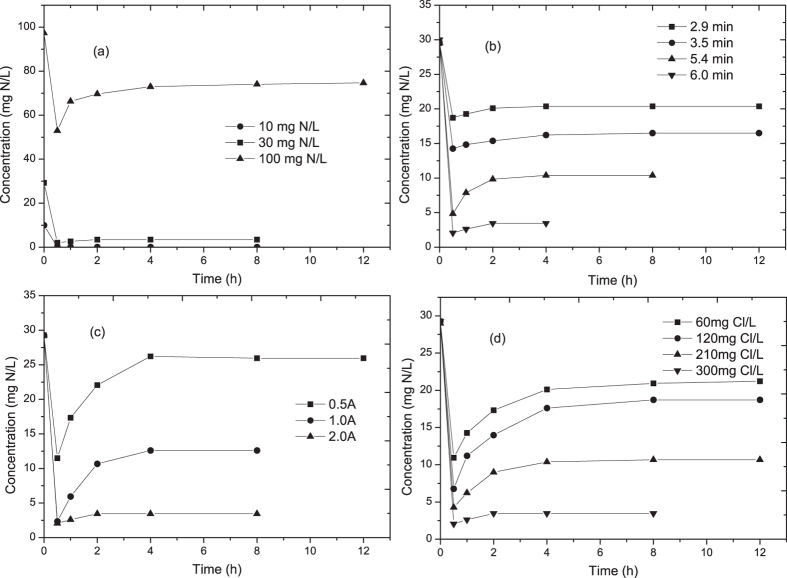
The effect of different factors on electrolytic removal of ammonia: (**a**) concentration of substrate; (**b**) HRT; (**c**) current (**d**) chloride.

For municipal wastewater or secondary effluent from municipal wastewater treatment plant, ammonia concentration fluctuated from several to tenths of milligrams per liter based on the source and treatment technologies. As shown in Fig. 4(a), 10–100 mg N/L ammonia was electrolyzed at a current of 2.0 A and 6.0 min HRT. A reverse peak was observed at 0.5 h due to fast adsorption and then adsorption-desorption equilibrium reached after electrolysis for 2–4 hours. The final ammonia concentrations in the effluent were ND, 3.4 and 74.7 mg N/L, respectively.

HRT was another main factor for the continuous electrolytic process^37^. As many articles showed, better removal efficiency generally could be achieved under higher HRT circumstance. Figure 4(b) showed that concentrations of ammonia were significantly decreased from 30.0 mg N/L to 20.4, 16.5, 10.4 and 3.4 mg N/L, respectively, when HRT varied from 2.9 to 3.5, 5.4 and 6.0 min gradually. Similarly, a reverse adsorption peak was observed due to fast adsorption, and 2–4 hours electrolysis led to an adsorption-desorption balance resulting in a stable ammonia concentration in the effluent. Moreover, linear relationship can be observed between HRT and ammonia removal efficiency. Longer HRT was beneficial for the electrolytic production of activate chlorine, thus led to a faster removal of ammonia in the solution as well as on the vermiculite. Compared with common particle electrode such as active carbon, vermiculite showed a higher adsorption affinity and faster ammonia removal rates under the same reaction conditions. Through the adsorption by vermiculite, the ammonia retention time was longer than HRT, which was beneficial for electrolytic removal of ammonia.

Figure 4(c) obviously showed the relationship between the ammonia removal efficiency and the applied current. When the current increased from 0.5 A to 1.0, 2.0 A, the effluent ammonia concentration decreased from 25.9 to 12.6, 3.4 mg N/L, respectively. Linear relationship was observed between current and ammonia removal efficiency. High current led to faster production of active chlorine, which was the main oxidant for ammonia removal. Thus a better removal efficiency of ammonia could be achieved under higher current.

Subsequently, the change-curve of ammonia concentration in terms of chloride concentration was exhibited in Fig. 4(d). Significant enhanced removal of ammonia nitrogen had been found in associated with higher chloride concentration. The ammonia removal ratio was 27.5% at initial chloride concentration of 60.0 mg Cl/L. Nevertheless, a remarkable ammonia removal percentage of 36.1%, 63.2%, 88.2% was observed when chloride concentration was increased to 120, 210, 300 mg Cl/L. Linear relationship between ammonia removal efficiency and chloride ion concentration can be calculated, which might be explained by the fact that faster active chlorine oxidation of ammonia was supported with plenty of chloride ions according to the eqs (1),(2),(3). Moreover, chloride ion (Cl^−^) was regenerated after the reaction with ammonia as shown in eqs (3),(4),(5), thus acted as a catalyst during electrolytic oxidation of ammonia.

**Figure 5:**
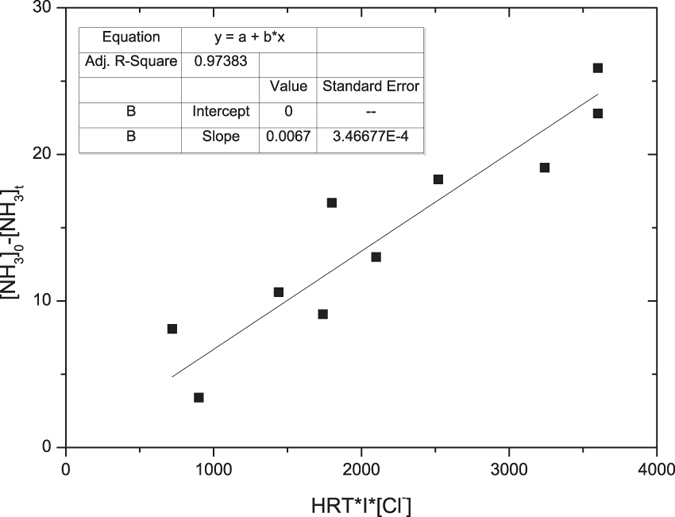
Relationship between ammonia removal and the product of HRT, current & chloride ion.

Considering the primary relationship between ammonia removal and related factors, primary data were fitted to a linear model as shown in Fig. 5. It was found that significant linear relationship existed between ammonia removal and the products of HRT, current and chloride ion concentration except the one with a low initial ammonia concentration of 10 mg N/L. The capacity of the electrolysis cell was not fully utilized under this condition. Generally speaking, the electrolytic removal of ammonia by using electrolysis can be simulated by the following eqn. (10), in which [NH_3_]_0_−[NH_3_]_t_ can be viewed as the ammonia removal capacity (ARC) and HRT, I, [Cl^−^] were hydraulic retention time, current and initial chloride ion concentration, respectively.


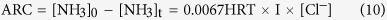


### Municipal wastewater treatment

**Figure 6:**
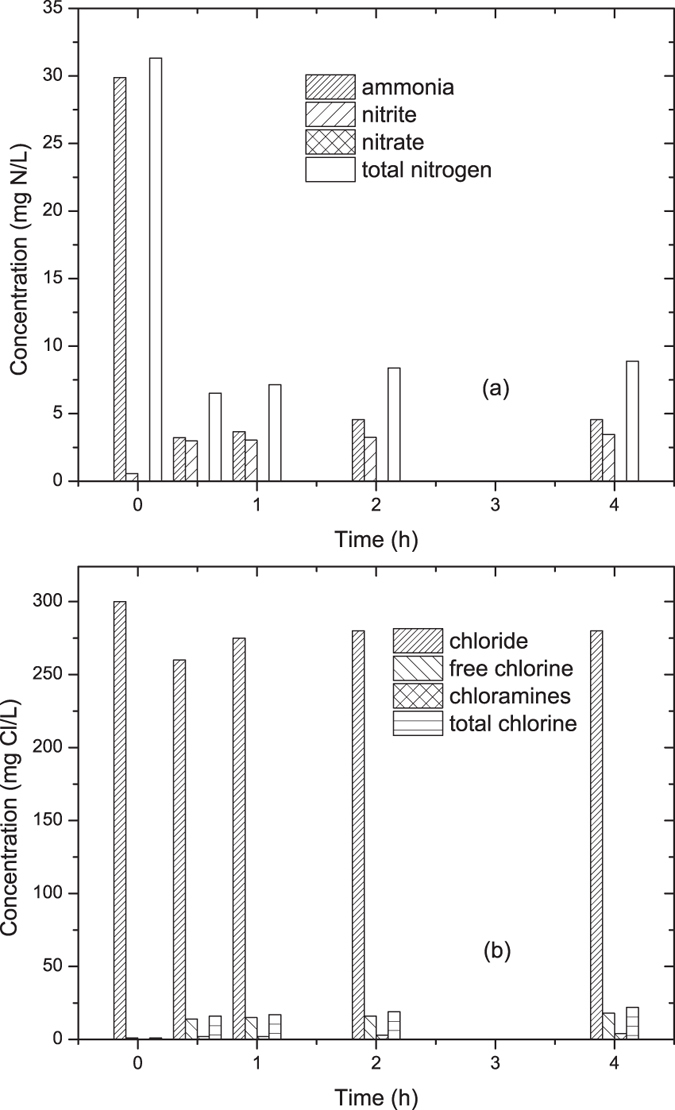
Electrolytic removal of ammonia from actual wastewater at 2.0 A current and 6.0 min HRT.

To further investigate the removal of ammonia and current efficiency by electrolysis cell packed with vermiculite, municipal wastewater was treated at a current of 2.0 A and 6.0 min HRT. As shown in Fig. 6, 29.9 mg N/L ammonia was reduced to 4.6 mg N/L, with a generation of 3.5 mg N/L nitrate. 85% ammonia removal was achieved, which proved that vermiculite was suitable for this process in the actual case. Both adsorption and electrolysis contributed for ammonia removal. Total nitrogen concentration reduced from 31.3 to 8.9 mg N/L, with residue nitrogen in the form of ammonia, nitrate, and tiny amount of nitrite and chloramines. 300 mg Cl/L chloride ion reduced to 280 mg Cl/L due to the generation of free chlorine and chloramines. According to eqn. (9), the current efficiency can be calculated to be 23%, which was lower than the synthetic wastewater. This might be explained by the reducing matters in the municipal wastewater, which consumed the oxidizing agents generated during electrolysis.

## Conclusions

This research evaluated the ammonia removal as well as current efficiency during electrolysis by using an electrolysis cells packed with vermiculite. 30.0 mg N/L ammonia can be reduced to 3.4 mg N/L under a current of 2.0 A and 6.0 min HRT, with a CE of 25%. Under similar conditions, a minor 2% reduction of CE was observed for the treatment of municipal wastewater. The packing of vermiculite increased the ammonia retention time in the electrochemical cell, thus improved the ammonia removal efficiency. Linear relationships were observed between ammonia removal and HRT, currents and chloride concentrations, respectively. The ARC of the electrolysis cell packed with vermiculite can be expressed as ARC = 0.0067HRT × I × [Cl^−^] within experimental conditions of 2.9–6.0 min HRT, 0.5–2.0 A current and 60–300 mg Cl/L chloride ion concentration.

## Materials and Methods

### Materials and reagents

All chemical reagents for the experiment (such as (NH_4_)_2_SO_4_, Na_2_SO_4_, NaCl, H_2_SO_4_, NaOH, N-(1-naphthyl)-ethylenediamine dihydrochloride, Nessler’s reagent, potassium sodium tartrate tetrahydrate etc) were purchased from Sinopharm Chemical Reagent Co., Ltd (Shanghai, China). Total and free chlorine reagent sets were bought from HACH Company (Loveland, CO, USA). Vermiculite was provided by Nan Yu Minerals Factory (Lingshou county, Hebei Province, China). Before use, vermiculite was washed by deionized water to remove possible salts and impurities during manufacture, and dried in the hot air oven at 50 °C.

### Characterization of vermiculite

The structure of the vermiculite was determined using an X-ray diffractometer (XRD, Bruker D8 Advance, German) with Cu-Kα radiation (40 kV, 40 mA). The as-received sample was scanned from 5° to 80° at 2°/min with a step size of 0.02°. Results were analyzed by the software Jade 6.5 equipped with a standard PDF2004 reference card. The nitrogen adsorption/desorption isotherms of vermiculite were determined by using a Physisorption Analyzer (Micromeritics ASAP 2020, USA) at 77.4 K. The specific surface area was obtained by Brunauer-Emmett–Teller (BET) method, while Density Functional Theory (DFT) method was employed to calculate the pore volume and diameter^38^.

### Experimental procedures for electrolytic removal of ammonia

The whole experiments were performed under continuous mode in an electrolysis reactor with the addition of vermiculite in the void space, prospecting for a better condition for enhancement of mass and electron transfer. Before installation, the stainless cathode and RuO_2_/Ti anode with the same dimension of 176 × 38 mm were immersed in 1% of dilute sulphuric acid to get rid of the attached impurities from electrodes surface. Real municipal or synthetic wastewater was driven by a peristaltic pump to pass through the electrolysis cell at a certain rate. 1 M NaOH or 0.5 M H_2_SO_4_ was used to adjust the pH of solution at 7.0 ± 0.5, which is close to the actual situation. The conductivity of the electrolyte was increased by adding Na_2_SO_4_. Samples were taken and filtered with 0.45 μm membrane filter periodically, and then measured according to standard method to monitor the transformation of nitrogen during electrolysis.

By using synthetic wastewater prepared with DI water and chemicals, the transformation of nitrogen and chloride elements were determined by analyze possible products during electrolysis, leading to the further understanding of mechanisms and calculation of current efficiency. The influencing of different factors (HRT 2.9–6.0 min, current 0.5–2.0 A, initial ammonia 30–100 mg N/L and chloride concentration 60–300 mg Cl/L) were investigated by a single factor strategy. One factor varied while the others were fixed at reference conditions of pH 7.0, 2.0 A current, 300 mg/L chloride and 6.0 min HRT, respectively. The effectiveness for ammonia removal in real wastewater was verified with secondary effluent from wastewater treatment plant at reference conditions.

The ammonia concentration was measured through Nessler’s method. Possible by-product of nitrite was measured with through N-(1-naphthyl)-ethylenediamine dihydrochloride spectrophotometric method. UV spectrophotometric method using spectrophotometer (Shimadzu UV-2600, Japan) was employed to determine nitrate concentration. Total nitrogen and pH were determined with pH meter (PHS-2C, Leici Company, Shanghai, China) and TOC/TN analyzer (Multi N/C 3100, Analytikjena Company, Germany), respectively. Free chlorine and total chlorine was measured through DPD ferrous titrimetric method. Chloride ion was measured through titration with silver nitrate solution.

## Additional Information

**How to cite this article:** Li, L. *et al*. Electrolytic ammonia removal and current efficiency by a vermiculite-packed electrochemical reactor. *Sci. Rep.* **7**, 41030; doi: 10.1038/srep41030 (2017).

**Publisher's note:** Springer Nature remains neutral with regard to jurisdictional claims in published maps and institutional affiliations.
